# Mapping longitudinally consistent intrinsic connectivity networks in macaque brain via longitudinal sparse dictionary learning

**DOI:** 10.1016/j.ibneur.2024.11.014

**Published:** 2024-12-04

**Authors:** Arif Hassan Zidan, Afrar Jahin, Yu Bao, Wei Zhang

**Affiliations:** a School of Computer and Cyber Sciences, Augusta University, United States; b Department of Graduate Psychology, James Madison University, United States; c Transdisciplinary Research Initiative in Inflammaging and Brain Aging (TRIBA), Augusta University, United States

**Keywords:** Longitudinal consistency, sparse dictionary learning, intrinsic connectivity network, macaque, resting-state fMRI, connectome scale

## Abstract

Mapping consistent longitudinal intrinsic connectivity networks (ICNs) is crucial for understanding brain functional development over various life stages. However, achieving consistent longitudinal ICNs has been challenging due to the lack of methodologies that maintain temporal consistency. To address this gap, we introduce an innovative approach named Longitudinal Sparse Dictionary Learning (LSDL). This method utilizes an additional Frobenius norm to bridge gaps between consecutive ICNs, facilitating the continuous transfer of the learned feature matrix to subsequent stages. Moreover, Matrix Backpropagation (MBP) is employed to effectively mitigate potential accumulative errors. Our validation results demonstrate that *LSDL* can successfully extract 21 consistent longitudinal ICNs in macaque brains. In comparative empirical evaluations with established methodologies, Fast Independent Component Analysis (FICA) and Sparse Dictionary Learning (SDL), *LSDL* showcases superior efficacy in modeling longitudinal functional Magnetic Resonance Imaging (fMRI) data. This approach opens new avenues for research into developmental brain dynamics and neurodegenerative disorders, providing a robust framework for tracking the evolution of brain connectivity over time.

## 1 Introduction

Intrinsic Connectivity Networks (ICNs) provide insights into brain region interactions, usually depicting variations in deoxyhemoglobin concentration through functional Magnetic Resonance Imaging (fMRI) (Bullmore and Sporns, 2009, Cui et al., 2022, Fiorenzato et al., 2019, Franzmeier et al., 2019, Li et al., 2019, Peng et al., 2023, Power et al., 2011, Stam, 2014, Van Den Heuvel and Pol, 2010, Yang et al., 2022). These ICNs, denoted as spatial features from fMRI data, directly pinpoint brain regions impacted by neurological disorders more effectively than time-series analysis (Holiga et al., 2019, Ou et al., 2021, Yan et al., 2019). Notably, during the COVID-19 pandemic, researchers observed impaired ICNs in patients with olfactory loss (Wingrove et al., 2023). A vital capability of ICNs is to detect the earliest signs of impairment before irreversible structural changes occur (Lurie et al., 2020, Reid et al., 2019, Filippi et al., 2019; Agarwal et al., 2023; Kim and Ogawa, 2012, Kim and Bandettini, 2023; Ou et al., 2021; Zamani Esfahlani et al., 2020). Recent advancements in brain imaging have successfully mapped ICNs in both human and primate brains. However, several critical questions still remain: How are ICNs established during infancy and the juvenile stages? When do ICNs start to show unique functionalities distinct between juvenile and adult phases? Are the functions of specific ICNs dependent on their pre-existing connections to other brain areas? Which brain regions continue to develop through adolescence? Investigating these aspects requires examining consistent longitudinal ICNs (i.e., ICNs derived from each longitudinal fMRI dataset) across developmental stages (Deen et al., 2017, Isik et al., 2017, Kanwisher, 2017, Pereira et al., 2018).

Unfortunately, mapping consistent longitudinal ICNs at a connectome scale is inherently challenging. It requires advanced technologies to create consistent connections across ICNs over time, alongside a deep understanding of the functional complexity and even hierarchical organization of developing ICNs (Deen et al., 2017, Giedd et al., 1999, Isik et al., 2017, Kanwisher, 2017). Most current research focused on specific and localized ICNs using region-of-interest (ROI) approaches (Bachevalier, 1994, Baron-Cohen et al., 2005, Beversdorf et al., 2005, Deco et al., 2011, Goldman-Rakic and Schwartz, 1982, Herting et al., 2018, Li et al., 2019, Lipska and Weinberger, 2002), and other studies have only addressed longitudinal changes within limited ICNs or singular networks like the default mode network (DMN) (Gao et al., 2009, Sacher and Staffeldt, 1974, Van Essen et al., 2011, Van Essen et al., 2001, Van Essen, 2004).

To address these challenges, we propose an innovative method, the Longitudinal Sparse Dictionary Learning (LSDL), based on our prior work (Zhang et al., 2017, Zhang et al., 2018a, Lv et al., 2015a, Lv et al., 2015b). Overall, building on the demonstrated efficacy of Sparse Dictionary Learning (SDL) in revealing ICNs at a connectome scale via resting-state fMRI (rsfMRI), which surpasses the performance of Independent Component Analysis (ICA) (Khalilullah et al., 2023, Zhang et al., 2018a), *LSDL* employs a dual approach: it incorporates an additional Frobenius norm to enhance longitudinal consistency and employs Matrix Backpropagation (MBP) to mitigate potential cumulative errors (Varoquaux et al., 2011, Wytock,, Zhang et al., 2018a, Zhang et al., 2018b, Lv et al., 2017, Trigeorgis et al., 2017, Beck and Teboulle, 2009, Zhao et al., 2015, Zhao et al., 2017). In particular, the application of *LSDL* on longitudinal fMRI of macaque brains has successfully identified 21 consistent longitudinal ICNs. Furthermore, extensive qualitative and quantitative validations emphasize the robustness of *LSDL*, unveiling continuous functional developments, particularly in areas like the visual and auditory cortex. Given the structural and functional parallels between macaque and human brains, studying longitudinal ICNs in macaques can illuminate aspects of early-stage brain functional evolution (Smyser et al., 2010, Zhang et al., 2017, McCormack et al., 2015, Zhang et al., 2019a, Zhang et al., 2019b) and improve our understanding of human brain development and architecture (Isik et al., 2017, Kanwisher, 2017; Pereira et al., 2018; Zhang et al., 2017, Zhang et al., 2018a, Zhang et al., 2018b; McCormack et al., 2015).

To summarize, this work not only advances our understanding of longitudinal development within ICNs but also pave the way for investigating neurodevelopmental, neurodegenerative, and psychiatric disorders through the innovative lens of consistent longitudinal ICNs.

## 2 Methods

This section is structured into four main sections: 1). Computational Framework of *LSDL*: This sub-section delves into the details of the *LSDL* computational framework. It covers methodologies for identifying consistent longitudinal features and strategies for minimizing accumulative error using Matrix Backpropagation (MBP) (refer to 2.1). 2). Data Acquisition and Pre-processing: We outline the extensive data acquisition and pre-processing procedures utilized in this study, ensuring a thorough understanding of our experimental setup (refer to 2.2). 3). Parameter Tuning: This section discusses our approach to parameter tuning, specifically designed to circumvent arbitrary manual settings, enhancing the reproducibility and robustness of our findings (refer to 2.3). 4). Identification of Consistent Longitudinal ICNs: We introduce the principles underlying our methodology for identifying consistent longitudinal ICNs, emphasizing the rigor and precision of our analytical techniques (refer to 2.4). Each section aims to provide a comprehensive understanding of our methods and results, fostering clarity and facilitating replication of our research.

### 2.1 Longitudinal sparse dictionary learning

**Fig. 1:**
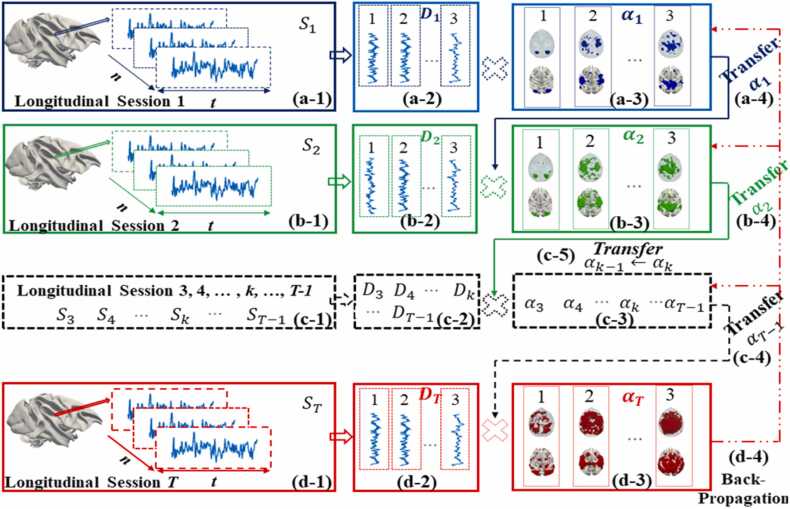
Illustration of the computational framework of *LSDL* for modeling longitudinal macaques rsfMRI data, based on subject-level. (a-1) The rsfMRI signals of macaque brains at the first-time point are extracted and re-organized as a 2D signal matrix; (a-2) we present three examples of a learned dictionary at the first time point; (a-3) similarly, we provide corresponding three examples of learned coefficient matrix at the first time point; (a-4) all learned features are transferred to the next step; (b-1), (b-2), (b-3) and (b-4) is presented the corresponding learning steps of second time point, similarly to previous steps (a-1), (a-2), (a-3) and (a-4), respectively; (c-1) the longitudinal data set from other points, e.g., ${\left\{S_{k}\right\}}_{k=1}^{T-1}\subseteq R^{t\times n}$ is collected and organized as 2D matrices; (c-2), (c-3) and (c-5) iteratively, we employ the model to implement the identification of longitudinal consistent features, via repeatedly transferring the learned features to the next step; $\alpha_{k-1}$ represents the feature matrix learned from the previous time point and $\alpha_{k}$ represents the current features that need to be learned; (c-4) transfer the learned feature matrix $\alpha_{T-1}$ to the final stage; (d-1), (d-2) and (d-3) is presented the corresponding learning steps of final time point, similarly to previous steps (b-1), (b-2) and (b-3), respectively; (d-4) After the final feature matrix/coefficient matrix has been learned, the MBP is performed to reduce the potential accumulative errors, caused by iterative transfer of feature matrices.

The computational framework of *LSDL* is depicted in Fig. 1. The primary innovation of *LSDL* lies in its capability to detect the longitudinal consistency of ICNs across various time points. Unlike traditional methods that independently analyze fMRI signals from independent time points, *LSDL* leverages an innovative technique entitled feature transfer to maintain the consistency in feature matrix across sequential scans. This methodology parallels the principles of deep learning (DL), where continuously transfer data is integral but can lead to cumulative errors. To address this challenge, we incorporate Matrix Backpropagation (MBP) (Gogtay et al., 2004, Zhang et al., 2017), a technique rooted in Non-negative Matrix Factorization (NMF) (Ding et al., 2005), specifically designed to minimize such errors. For a detailed explanation of this innovative approach, please refer to the legend accompanying Fig. 1.

### 2.2 Longitudinal feature transfer and matrix backpropagation

Consider the set of longitudinal rsfMRI signal matrices from the macaque brains, represented as ${\left\{S_{k}\right\}}_{k=1}^{T}\subseteq R^{t\times n}$, k=1,2,⋯, where k is the index for each time point and T is the total number of rsfMRI scan sessions for each individual. In addition, *n* is the voxel number of whole brain space, and *t* represents the temporal dimension of each signal matrix, e.g., $S_{k}$. The objective of *LSDL* is to decompose a dictionary matrix (i.e., weight/mixing matrix) from the initial scan session, denoted as $D_{1}\in R^{t\times m}$, and a corresponding sparse coefficient matrix, denoted as $\alpha_{1}\in R^{m\times n}$:

(1) $$F\left(D_{1},\alpha_{1}\right)=\frac{1}{2}{\|S_{1}-D_{1}\alpha_{1}\|}_{F}^{2}+\lambda {\|\alpha_{1}\|}_{1}$$

where $S_{1}$ is the input signal matrix of rsfMRI at the first time point. In addition, λ represents the sparse trade-off. Eq. (1) is the same as the objective function of conventional SDL (Lv et al., 2015a, Lv et al., 2015b, Zhang et al., 2018a, Zhang et al., 2018b, Varoquaux et al., 2011). Notably, m represents the number of dictionary atoms, encapsulating the dimensionality of the feature space.

Furthermore, the objective function governing other time points shown as below:

(2) $$F\left(D_{k},\alpha_{k}\right)=\frac{1}{2}{\|S_{k}-D_{k}\alpha_{k}\|}_{F}^{2}+\lambda {\|\alpha_{k}\|}_{1}+\frac{1}{2}\mu {\|\alpha_{k-1}-\alpha_{k}\|}_{F}^{2}$$

where $S_{k}$, $D_{k}$ and $\alpha_{k}$, ∀k>1, is the input signal matrix of rsfMRI, dictionary and coefficient matrix, at k time point, respectively; $\alpha_{k-1}$ is the learned coefficient matrix from the last time point, transferred from the previous time point session (*k*-1 time point). This framework aims to decompose each signal matrix $S_{k}$ into the product of $D_{k}$ and $\alpha_{k}$, effectively representing each matrix as a combination of dictionary atoms and their corresponding activation patterns, which collectively constitute various ICNs. More importantly, the task involves creating a coefficient matrix that captures the essence of the ICNs over time by maintaining consistency across all temporal segments via an additional Frobenius norm, ${\|\alpha_{k-1}-\alpha_{k}\|}_{F}^{2}$, in Eq. (2) to bridge the gap between adjacent longitudinal feature matrices, facilitating the consistent identification of ICNs. By iteratively applying Eq. (2), *LSDL* ensures the preservation of longitudinal consistency of the feature matrices across all time points.

Moreover, the iterative transfer the matrices can result in the potential accumulative error; to reduce the possible accumulative error, based on some previous works (Trigeorgis et al., 2017), a non-negative MBP (NMBP) is proposed:

(3) $${\hat{\alpha }}_{k}⟵\alpha_{k},\;k=T\;{\hat{\alpha }}_{k}⟵D_{k+1}{\hat{\alpha }}_{k+1},\;k<T\;$$

In Eq. (3), if *k* is equal to final time point *T*, we directly set $\alpha_{k}$ as ${\hat{\alpha }}_{k}$; otherwise, if *k* is smaller than *T,* we utilize $D_{k+1}{\hat{\alpha }}_{k+1}$ to represent ${\hat{\alpha }}_{k}$. And $D_{k+1}$ is denoted as $D_{k+1}⟵\psi^{†}S_{k}{{\hat{\alpha }}_{k}}^{†}$; the operator ${\left[\;∙\;\right]}^{†}$ denotes the Moore-Penrose pseudo-inverse of an input matrix. ψ is defined as the product of a series of longitudinal dictionaries as $\prod_{i=1}^{k-1}D_{i}$. We can update ${\hat{\alpha }}_{k}$ by:

(4) $${\hat{\alpha }}_{k}⟵{\hat{\alpha }}_{k}⊙\sqrt{\frac{{\left[\psi^{T}S_{k}\right]}^{+}+{\left[\psi^{T}\psi \right]}^{-}{\hat{\alpha }}_{k}}{{\left[\psi^{T}S_{k}\right]}^{-}+{\left[\psi^{T}\psi \right]}^{+}{\hat{\alpha }}_{k}}}$$

where ${\left[∙\right]}^{+}$ represents the selection of positive values of input matrix, and ${\left[∙\right]}^{-}$ indicates the selection of negative values of input matrix.

The original MBP methods described by (3), (4) require non-negative or semi non-negative values (Trigeorgis et al., 2017). The tackling of negative values in the feature matrices, e.g., ${\left\{\alpha_{k}\right\}}_{k=1}^{T}\subseteq R^{t\times L}$, which usually represent non-activated areas within the ICNs, presents a unique challenge for matrix-based methods that typically require non-negative values. To address this issue, we propose a novel approach where each learned coefficient matrix, e.g., ${\hat{\alpha }}_{k}$, is split into positive and negative components, denoted as ${\hat{\alpha }}_{k}^{+}$ and ${\hat{\alpha }}_{k}^{-}$, respectively. This separation allows us to conform to non-negative matrix requirements by handling the positive and absolute values of negative components distinctly. The negative components of ${\hat{\alpha }}_{k}$ are transformed into absolute values, noted as $\left|{\hat{\alpha }}_{k}^{-}\right|$. Then, the MBP is applied separately to ${\hat{\alpha }}_{k}^{+}$ and $\left|{\tilde{\alpha }}_{k}^{-}\right|$, ensuring that the backpropagation conforms to non-negative constraints. The updated negative components $\left|{\tilde{\alpha }}_{k}^{-}\right|$ are converted back to their original negative state by multiplying by −1, resulting in $-\left|{\tilde{\alpha }}_{k}^{-}\right|$. This approach ensures that the integrity and representational accuracy of the original feature matrices are maintained, facilitating the preservation of essential information regarding non-activated areas within ICNs. The details of pseudo-code of proposed core algorithm are shown as below:

Core Algorithm: Longitudinal Sparse Dictionary Learning (LSDL)

**Table:** 

### 2.3 Data acquisition and preprocessing

Rhesus macaques (Macaca mulatta) are a crucial model in neuroscience research due to their close genetic and behavioral similarities to humans (Zhang et al., 2017). This study involved four subjects from a longitudinal cohort at the Yerkes National Primate Research Center (YNPRC) at Emory University, located in Lawrenceville, Georgia. These subjects, scanned at 3, 6, 12, and 18 months of age, were part of a broader investigation into the developmental trajectories of socially raised macaques (Zhang et al., 2017, Zhang et al., 2019a). Raised in large social groups across various social hierarchies, they were nourished with a diet rich in seasonal fruits, vegetables, and high-fiber, low-fat primate chow, supplemented with enrichment items. Water was provided ad libitum. The ethical considerations of this study were rigorously maintained, adhering to the Animal Welfare Act and the U.S. Department of Health and Human Services guidelines, with approval from the Emory University Institutional Animal Care and Use Committee (IACUC) (Zhang et al., 2017, Zhang et al., 2019a). Imaging was conducted using a 3 T Siemens Trio scanner equipped with an 8-channel knee coil, at YNPRC. The macaques were anesthetized with isoflurane (0.8–1 %) to minimize the impact on functional connectivity and positioned using a customized head holder to prevent motion artifacts (Zhang et al., 2017, Zhang et al., 2018a, Zhang et al., 2019a). All were intubated and hydrated intravenously during scans and warmed with an MRI-compatible heating pad. Post-recovery, they were returned to their familial social groups.

Specifically, scanning parameters included an echo planar imaging (EPI) sequence with a repetition time (TR) of 2060 msec, an echo time (TE) of 25 msec, and a resolution of 1.5 × 1.5 × 1.5 mm^3^. Each session consisted of two 15-minute scans, yielding 400 time points per scan. Data preprocessing involved skull removal, motion correction, slice timing correction, spatial smoothing, temporal pre-whitening, global drift removal, and band-pass filtering, using FSL toolkit and in-house tools (Zhang et al., 2017, Bassett et al., 2008). The INIA19 macaque brain atlas was used as a reference for all detected ICNs, which were aligned to the atlas using FSL-FLIRT to ensure the identification of longitudinally consistent ICNs across all scans and ages (Zhang et al., 2017, Rohlfing et al., 2012). Each individual brain mask was used to extract the fMRI signals, ensuring personalized processing. The volume matrix was 85 × 104 × 65, encompassing 34,000–45,000 voxels per mask, tailored to the unique anatomical features of each subject (Zhang et al., 2017, McCormack et al., 2015).

Notably, acquiring fMRI data from animals presents unique challenges that differ significantly from human data collection (Balezeau et al., 2021). For instance, even primates cannot follow instructions as accurately as humans, often requiring anesthesia during fMRI sessions. Although the cost of raising and maintaining primates for research is exceptionally high, small sample sizes are not uncommon in impactful research involving non-human primates (Nakahara et al., 2002, Nelissen and Vanduffel, 2011).

### 2.4 Hyperparameter and parameter tuning

To optimally determine crucial parameters in *LSDL*, such as dictionary size, sparse trade-off, and parameters controlling the similarity between adjacent coefficient matrices, we utilize multiple techniques for parameter tuning, guided by our previous research (Zhang et al., 2017, Zhang et al., 2019a). Initially, we utilize a technique known as the rank estimator to determine the dictionary size. This rank estimator (Zhang et al., 2017, Varoquaux et al., 2011, Liu et al., 2010) advances the determination of potentially optimal dictionary size, which corresponds to the input dictionary size used in the LASSO (Lv et al., 2015a, Lv et al., 2015b, Lv et al., 2017, Zhang et al., 2017, Zhang et al., 2018a). This approach involves accurately estimating the rank of each individual fMRI signal matrix, which informs the dictionary size and ensures the adequacy to capture the inherent complexity of the input data such as ${\left\{S_{k}\right\}}_{k=1}^{T}\subseteq R^{t\times n}$. The rank estimator utilizes a rank-revealing method based on Orthogonal Decomposition, specifically QR factorization (Katsikis et al., 2021, Wen et al., 2012). Initially, the method determines the estimated rank of each signal matrix $S_{k}$. This initial rank helps guide the iterative calculation to find the optimal rank for the input matrix. If the initial estimated rank is found to be larger than the optimal rank, the algorithm examines the diagonal elements of the upper-triangular matrix R resulting from the QR factorization of $S_{k}$ (Wen et al., 2012). This step is crucial to ensure that any redundancy in the data representation is minimized, thus enhancing the sparsity and effectiveness of the dictionary learning process.

In addition, we can confirm the optimal size of $S_{k}$ through QR factorization. During this phase, the magnitudes of the diagonal elements of R should ideally show a non-increasing trend (Katsikis et al., 2021). This condition indicates that the matrix dimensions are correctly estimated, optimizing the computational efficiency and accuracy of the model. To finalize the rank determination, the algorithm employs thresholding values specified in Eqs. (5) and (6), which solidify the rank decision by establishing clear cutoffs (Liu et al., 2010) based on the matrix's diagonal line, denoted as $R_{\mathrm{ii}}$. From this diagonal, $d_{i}$ and $d_{i+1}$ are computed to quantitatively describe the rank structure of $S_{k}$. These vectors assist in the final determination of the dictionary size and composition, ensuring that the learned features are both representative and efficient in capturing the underlying patterns of the fMRI data.

(5) $$d_{i}=\left|R_{\mathrm{ii}}\right|r_{i}=\frac{d_{i}}{d_{i+1}}$$

and then examine the value:

(6) $$\varsigma =\frac{\left(m-1\right)r(p)}{\sum_{i\neq p}r_{i}}$$

where r(p) is the maximum element of the vector $r_{i}$ (with the largest index *p* if the maximum value is not unique). In our current implementation, we reset the rank estimated *r* top once ς>2, and this adjustment can be successfully done only once (Katsikis et al., 2021). By using the rank-revealing, we can set the initial number of components, i.e., ICNs, the same as input matrix, e.g., 400.

Moreover, another important parameter denoted as the sparse trade-off can be estimated via Rose algorithm. According to the theoretical research, we aim to optimize the selection of a crucial parameter λ, that is the sparse trade-off. Based on the theorem in research works, if assume μ to be 0, we have an equivalent format of a min-max problem, e.g., dual format for a single time points of Eq. (4) here:

(7) $$\min_{X\in R^{t\times n}}{\;\max}_{{\|Z\|}_{\infty }}L\left(S,D,\alpha ,Z\right)=\frac{1}{2}{\|S-D\alpha \|}^{2}+\lambda <X\alpha ,Z>$$

The linear system can be organized as:

(8) $$XX^{T}Z=\mathrm{XS}$$

Eq. (8) has a unique solution, denoted by $\hat{Z}$. Let

(9) $$\lambda_{\max}={\|\hat{Z}\|}_{\infty }$$

Using the Rose algorithm provided in [41], let $XX^{T}$ be the Hessian matrix, and then we can estimate the maximal value of λ. An attenuation series has been introduced in the literature (Liu et al., 2010) as$\;{[10}^{-3},\;10^{-2},10^{-1}]$ that is adopted to determine the value of λ, i.e., $\lambda =10^{-p}\times \lambda_{\max}$, p=1,2,3,⋯, and$\;{\lambda =\lambda_{\max}\times 10}^{-3}$. Therefore, we set $\lambda =10^{-3}\times \lambda_{\max}$. By the estimation using Eq. (9), we have λ=0.11.

**Fig. 2:**
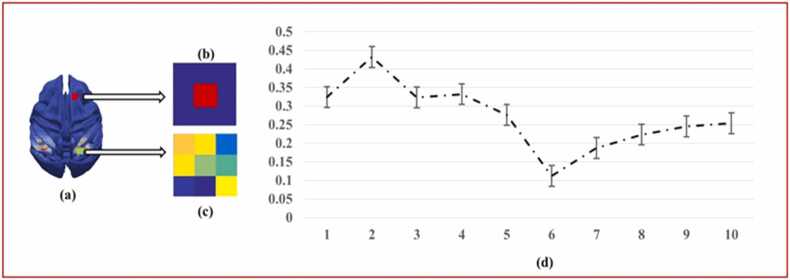
The description of framework for our proposed method on identification of threshold to binarize identified ICNs. (a) an identified ICN from coefficient matrix is visualized, and two different voxels denoted via (10), (11) are presented as red, green square, respectively; (b) an example of the noisy voxel, represented as ${\mathrm{Pixel}}_{1}$ in Eq. (10), is described as a red square, and its surrounding area is empty; (c) an example of meaningful voxel, denoted as Eq. (11), is shown as the central green square and its neighbor voxels are non-zero; (d) we calculate the average ratio between these two kinds of voxels, based on the identified ICNs extracted from the coefficient matrices of all subjects; the numbers of 1–10 represent the corresponding value of μ, from 0.1 to 1.0; obviously, the 0.60 is the optimal value for μ, which can provide the less noisy voxels.

Furthermore, to empirically determine the parameter μ which controls the similarity between features from adjacent time points, we conduct an experiment spanning a range of μ values from 0.1 to 1.0. Our method involves analyzing all voxels within the identified coefficient matrices, which represent the ICNs or feature matrices. Specifically, we categorize voxels into two categories based on their contribution to the signal quality: noisy voxels and meaningful voxels. Noisy voxels are identified through Eq. (10), which likely include background noise or non-neuronal fluctuations. Conversely, meaningful voxels, representing significant neural activity, are identified using Eq. (11) and are visually represented by red squares in Fig. 2. This distinction is critical for evaluating the quality and reliability of the ICNs extracted by our method. Specifically, two categorizations of voxels construct two corresponding sets, and the mathematic description is:

(10) $${\mathrm{Voxel}}_{1}:=\left\{x_{i},\;x_{j}|i\neq j,B\left(x_{i},\varepsilon \right)\cap B\left(x_{j},\varepsilon \right)=\emptyset \right\}\;$$

(11) $${\mathrm{Voxel}}_{2}:=\left\{y_{i},\;y_{j}|i\neq j,\;B\left(y_{i},\varepsilon \right)\cap B\left(y_{j},\varepsilon \right)\neq \emptyset \right\}\;$$

where $B\left(∙,\varepsilon \right)$ represents a given open cube, and radius ∀ε>0 (Wheeden, 2015); for $B\left(∙,\varepsilon \right)$, ∙ is denoted as the center of the given cube (Mantini et al., 2013). By examining the ratio of two kinds of voxels, the optimal μ can be decided in this study. The following figure briefly describe the method to determine the parameter μ.

Moreover, to further eliminate arbitrary within identified ICNs via *LSDL*, Fast Independent Component Analysis (FICA) (Calhoun et al., 2001, Khalilullah et al., 2023), and Online Dictionary Learning (ODL) (Lv et al., 2015a, Lv et al., 2015b), we standardize all intensities of identified ICNs using Z-score. Specifically, we convert these intensities into Z-scores, following the methodologies described in (Zhang et al., 2017, Zhang et al., 2018a, Lv et al., 2015a, Lv et al., 2015b, Varoquaux et al., 2011). These Z-scores are then binarized to facilitate voxel counting, which is a critical step in our subsequent validation processes.

Besides, we propose a novel voxel count technique, which is a crucial metric for assessing longitudinal variations across different time sessions (Lv et al., 2015a, Lv et al., 2015b, Howell et al., 2016, Caviness et al., 1996). In particular, the voxel count technique comprehensively considers adjacent voxels’ intensity and determines the threshold to facilitate the calculation of area, e.g., the number of voxels, in each identified ICN. Benefitting from voxel count, we can differentiate the variation of area in each ICN.

### 2.5 The principle to detect consistent longitudinal ICNs in macaque brain

In our prior studies (Zhang et al., 2017, Zhang et al., 2018a; Zhang et al., 2019a, 2019b), we have effectively documented 70 intrinsic connectivity network (ICN) templates for the macaque brain. Additionally, we provide individual longitudinal ICNs corresponding to these templates, enhancing our understanding of their consistent longitudinal development. We employed ICA, SDL, and *LSDL*, following recommended parameters (Lv et al., 2015a, Lv et al., 2015b, Lv et al., 2017, Zhang et al., 2017, Zhang et al., 2018a, Zhang et al., 2018b; Beck and Teboulle, 2009, Varoquaux et al., 2011, Wytock,), to detect longitudinally consistent ICNs. Specifically, FICA (Calhoun et al., 2001, Khalilullah et al., 2023) utilizes a single vital parameter—the number of potential independent components—to automatically estimate parameters and determine the number of components. For ODL, Lv et al. recommended parameters include a dictionary size of 400 and a sparse trade-off of 0.15 (Lv et al., 2015a, Lv et al., 2015b, Lv et al., 2017).

Our rigorous principle for determining longitudinal consistency ensures that all identified ICNs across all time points exhibit monotonous variance. We define monotonicity qualitatively, where the regions of an ICN should expand beyond those identified in previous time points, aligning with adolescent neural developments (Deen et al., 2017, Isik et al., 2017, Kanwisher, 2017, Pereira et al., 2018, Malkova et al., 2006, Knickmeyer et al., 2008, Knickmeyer et al., 2010, Machado and Bachevalier, 2003). Quantitatively, the voxel count within an ICN should consistently increase across time points, using a predetermined threshold (Malkova et al., 2006, Knickmeyer et al., 2008, Knickmeyer et al., 2010, Machado and Bachevalier, 2003).

In the following sections, we present and examine results based on these qualitative and quantitative criteria. Using prior research (McCormack et al., 2015, Zhang et al., 2017), we align identified ICNs via FICA, ODL, and *LSDL* with established macaque brain ICN templates (Machado and Bachevalier, 2003, McCormack et al., 2015, Zhang et al., 2017; Bosman et al., 2012). This alignment uses our rigorous principle to pinpoint potential longitudinally consistent ICNs. We offer a comprehensive qualitative presentation to validate the three methods' performance, focusing on longitudinal consistency, supplemented by an overarching quantitative analysis to affirm the methods' efficacy. Further details and explanations are provided in the subsequent sections.

## 3 Results

We employ two different computational frameworks: ME-ICA & DELMAR versus DELMAR/Denoise/Mapping to investigate the hierarchical organization of BCNs and their reproducibility from MBME fMRI. As discussed, in following sections, we hope to prove multiple hypotheses raised in the Introduction section.

### 3.1 Experimental comparison of LSDL with FICA and SDL

**Fig. 3:**
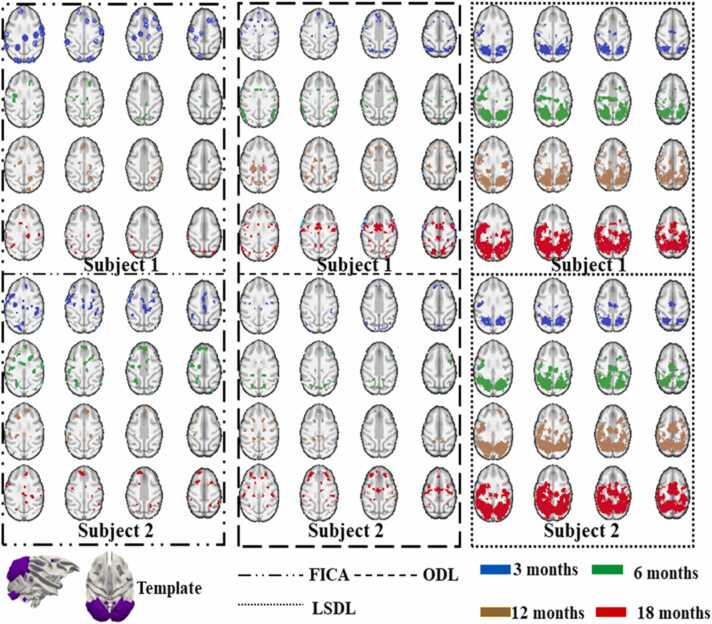
This figure provides a qualitative comparison from two subjects of ICA, ODL and *LSDL* based on the identified template of visual area (Zhang et al., 2017, Miyamoto et al., 2013, Nakahara et al., 2002, Nelissen et al., 2005, Nelissen and Vanduffel, 2011). The different dash lines represent the results obtained by different methods. And different colors represent the identified ICNs from different time points. By qualitative observation, the results from *LSDL* demonstrate the longitudinal consistency. The activation regions of ICNs are becoming larger and stronger through all time points. However, the ICNs identified via FICA and ODL are not very consistent. The identified ICNs are varied from initial time points to the final time points. Obviously, for example, some identified ICNs via ICA and ODL, occupy the larger brain regions, but some revealed regions become smaller at the final stage.

**Fig. 4:**
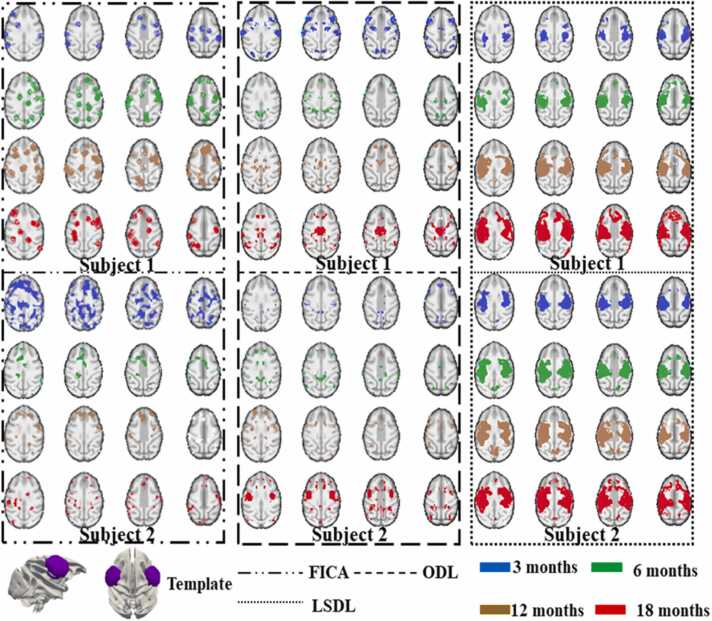
This figure provides a qualitative comparison from two subjects of ICA, ODL, and *LSDL* based on the identified template of auditory area (Zhang et al., 2017, Miyamoto et al., 2013, Nakahara et al., 2002, Nelissen et al., 2005, Nelissen and Vanduffel, 2011). By qualitative observation, the results from *LSDL* demonstrate the longitudinal consistency. The notations are the same or similar as those discussed in Fig. 3. Similarly, it is easy to observe the longitudinal consistent ICNs identified by LSDL. Meanwhile, both FICA and ODL cannot successfully identify the longitudinal consistent features. And the ICNs obtained by *LSDL* demonstrate that the monotonous development of longitudinal ICNs which also satisfy the reports of juvenile developmental brains (Zhang et al., 2017, Miyamoto et al., 2013, Nakahara et al., 2002, Nelissen et al., 2005, Nelissen and Vanduffel, 2011).

**Fig. 5:**
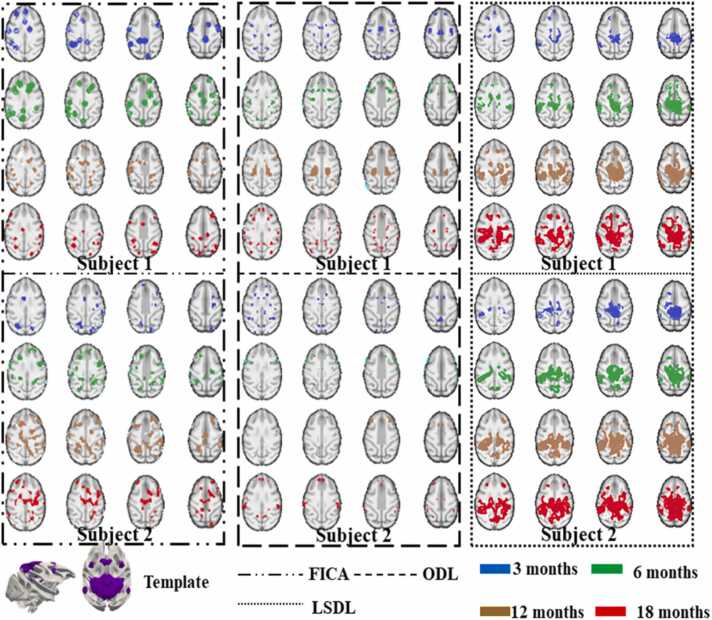
This figure provides the qualitative comparison from two subjects of ICA, ODL and *LSDL* based on the identified template of DMN (Zhang et al., 2017, Zhang et al., 2018a, Zhang et al., 2018b). The notations are the same or similar as those discussed in Fig. 3. Like other qualitative examples, the identified longitudinal DMNs via *LSDL* are gradually stronger than previous time points. However, the results obtained by FICA and ODL cannot successfully demonstrate the longitudinal consistency/monotonicity.

To validate the efficacy of the proposed *LSDL*, we conducted comparative analyses using two representative peer methods: FICA and ODL (Calhoun et al., 2001; Khalilullah et al., 2023; Lv et al., 2015a, Lv et al., 2015b, Lv et al., 2017). These methods are widely recognized as efficient, data-driven approaches for modeling fMRI signals (Zhang et al., 2017, Vanduffel et al., 2014, Miyamoto et al., 2013, Nakahara et al., 2002, Nelissen et al., 2005, Nelissen and Vanduffel, 2011). Utilizing the recommended parameters from prior research (Zhang et al., 2017, Zhang et al., 2018a, Zhang et al., 2018b Miyamoto et al., 2013; Nakahara et al., 2002), we applied FICA and ODL to perform identical ICN identifications. Our previous study (Zhang et al., 2017, Zhang et al., 2018a, Zhang et al., 2019a) have documented 70 ICN templates at the connectome scale for macaque brains. Initially, we aligned the ICNs identified by FICA, ODL, and *LSDL* with these previously identified templates. Given that *LSDL* is specifically designed to detect longitudinally consistent features, we implemented a rigorous validation framework to assess the longitudinal trajectories of ICNs and compare the effectiveness of the three methods. This evaluation framework aims to highlight the robustness of *LSDL* in maintaining longitudinal consistency across multiple temporal stages. In the ensuing figures, we present a qualitative comparison of the three methodologies using three illustrative templates: the visual area, auditory area, and the Default Mode Network (DMN). These comparisons are intended to demonstrate the distinct capabilities of each method in capturing and consistently tracking the dynamic changes within specific ICNs over time in Fig. 3, Fig. 4, Fig. 5.

In addition, following the presentation of the three examples, we offer additional qualitative comparisons through detailed visualizations and slices of identified ICNs, using the different methodologies applied. Below are links to the visual outputs for each method, providing a deeper insight into the ICNs identified in the four macaque subjects:

1).http://hafni.cs.uga.edu/LSDL_Project/ICA/MonkeyBrain_FastICA_Subject_1_LongitudinalVarianceComponentTemplatesMap_presentation.html

2).http://hafni.cs.uga.edu/LSDL_Project/ICA/MonkeyBrain_FastICA_Subject_2_LongitudinalVarianceComponentTemplatesMap_presentation.html

3).http://hafni.cs.uga.edu/LSDL_Project/ICA/MonkeyBrain_FastICA_Subject_3_LongitudinalVarianceComponentTemplatesMap_presentation.html

4).http://hafni.cs.uga.edu/LSDL_Project/ICA/MonkeyBrain_FastICA_Subject_4_LongitudinalVarianceComponentTemplatesMap_presentation.html

Moreover, for further qualitative comparison using ODL method, here are the links showcasing the ICNs identified in the four macaque subjects involved in our study:

1).http://hafni.cs.uga.edu/LSDL_Project/SDL/MonkeyBrain_ODL_Subject_1_LongitudinalVarianceComponentTemplatesMap_presentation.html

2).http://hafni.cs.uga.edu/LSDL_Project/SDL/MonkeyBrain_ODL_Subject_2_LongitudinalVarianceComponentTemplatesMap_presentation.html

3).http://hafni.cs.uga.edu/LSDL_Project/SDL/MonkeyBrain_ODL_Subject_3_LongitudinalVarianceComponentTemplatesMap_presentation.html

4).http://hafni.cs.uga.edu/LSDL_Project/SDL/MonkeyBrain_ODL_Subject_4_LongitudinalVarianceComponentTemplatesMap_presentation.html

Besides, for a thorough qualitative comparison using the *LSDL* method, here are the links showcasing the ICNs identified in the four macaque subjects involved in our study:

1).http://hafni.cs.uga.edu/LSDL_Project/LSDL/MonkeyBrain_LSDL_Subject_1_LongitudinalVarianceComponentTemplatesMap_presentation.html

2).http://hafni.cs.uga.edu/LSDL_Project/LSDL/MonkeyBrain_LSDL_Subject_2_LongitudinalVarianceComponentTemplatesMap_presentation.html

3).http://hafni.cs.uga.edu/LSDL_Project/LSDL/MonkeyBrain_LSDL_Subject_3_LongitudinalVarianceComponentTemplatesMap_presentation.html

4).http://hafni.cs.uga.edu/LSDL_Project/LSDL/MonkeyBrain_LSDL_Subject_4_LongitudinalVarianceComponentTemplatesMap_presentation.html

**Fig. 6:**
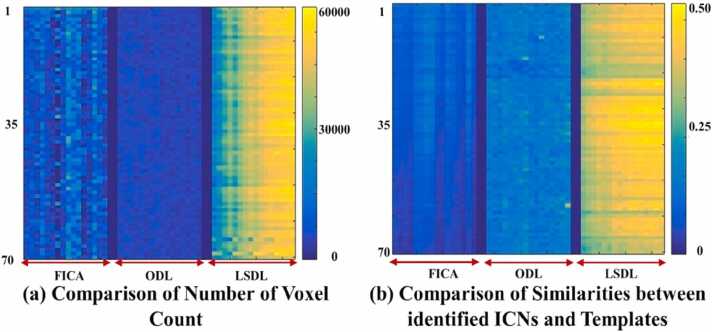
The overall and comprehensive quantitative comparison of ICA, ODL, and *LSDL*, based on 10 repeated experiments. These two subfigures (a) and (b) present the quantitative analyses of longitudinal voxel count, shown in (a) as well as the similarities between each identified ICNs and original templates, presented in (b). For subfigures (a) and (b), each column represents the corresponding average result calculated from a single time point from a macaque subject; all time points are organized from 3 months to 18 months. Obviously, by the observation, the results achieved by *LSDL* demonstrate the efficient performance to identify the longitudinal consistent ICNs from 3 months to 18 months, presented from the left to the right column.

Lastly, to further validate the proposed *LSDL* method, we conducted ten independent trials for each method, such as FICA, ODL, and *LSDL* to ensure a consistent quantitative comparison. The results are graphically presented in Fig. 6, which comprises several parts detailing our findings. In particular, Fig. 6(a) showcases the longitudinal voxel count analysis. To standardize measurements and avoid arbitrary comparisons, all identified ICNs were converted into Z-scores as per protocols in references (Zhang et al., 2017, Zhang et al., 2018a, Zhang et al., 2019a, Lv et al., 2017). We then applied a threshold of 0.10 (Zhang et al., 2017, Zhang et al., 2018a, Zhang et al., 2019a, Lv et al., 2017) to count the number of voxels for each identified ICN. These voxel counts, organized from ages 3 months to 18 months across all subjects, demonstrate a consistent increase in voxel numbers over time with *LSDL*, indicating robust feature detection. In contrast, the voxel counts from ICAs and ODL varied significantly, lacking a clear pattern of consistency or monotonic increase. Meanwhile, Fig. 6(b) utilized the Hausdorff metric (Zhang et al., 2017) to evaluate the similarity between the identified longitudinal ICNs and predefined templates. The similarities, arranged by columns for each age from 3 to 18 months, showed increasing congruence over time when using *LSDL*, affirming its effectiveness in tracking longitudinal development. This pattern was not as pronounced with FICA, and ODL exhibited significant longitudinal variations, with FICA showing a peculiar peak in similarity at the second time point only. Importantly, we leverage techniques such as voxel counting, which allows us to quantify the number of voxels that surpass a defined threshold (illustrated in Fig. 6(a)), and spatial similarity metrics (illustrated in Fig. 6(b)) that align the identified individual ICNs with established templates (Zhang et al., 2017). These quantitative methods offer robust, precise insights, even in smaller-scale studies, making them particularly well-suited to our investigation. By employing these approaches, we can efficiently and effectively validate the individual ICNs without the need for large, resource-intensive datasets (Zhang et al., 2017). To summarize, these results clearly highlight the advanced capability of *LSDL* in identifying robust and consistent ICNs longitudinally, compared to the traditional methods of FICA and ODL. The results further reinforce *LSDL* as an advanced technology in longitudinal brain connectivity studies.

### 3.2 Identified consistent longitudinal ICNs in macaque brains

In this section, we present 21 consistent longitudinal ICNs via *LSDL*. These ICNs showcase changes in activation intensity across developmental stages, indicating either intensification or diminution relative to earlier time points. This observation confirms our method's ability to capture the dynamic evolution of ICNs across different ages in macaques.

**Fig. 7:**
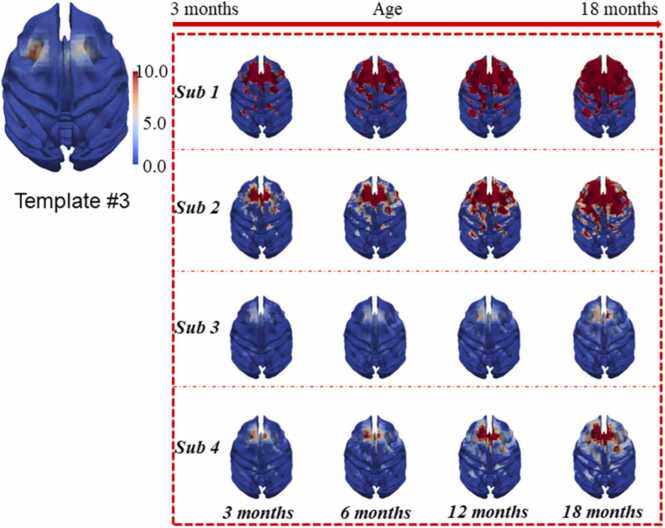
The presentation of an example of longitudinal consistent ICN #3, via mapping the detected ICNs to the cortical surface of macaque brain, to provide an overall qualitative observation; by the mapping to the surface, it is easier to observe the longitudinal developments of juvenile ICNs of macaques; the regions mapped to the cortical surface of ICNs is enlarging through all time points.

**Fig. 8:**
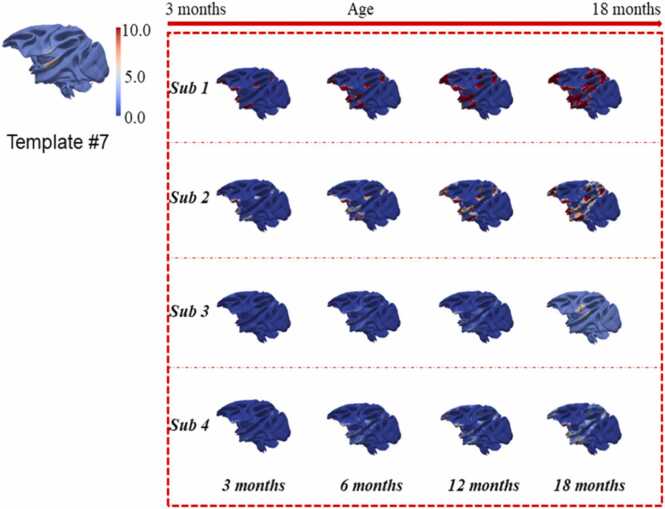
The presentation of another example of longitudinal consistent ICN #7, via mapping the detected spatial networks to the cortical surface of macaque, to provide an overall observation; it is obvious to observe the longitudinal developments of juvenile ICNs of macaques; the regions mapped to the cortical surface of ICNs is developing through all time points.

Notably, the effectiveness of LSDL in mapping these changes provides substantial evidence concerning the developmental trajectory of juvenile primate brains. Primarily, our findings contribute significantly to understanding how primates rely on their visual and auditory systems for environmental interaction, which aligns with numerous prior studies that have focused on the visual cortex and related neural networks (Howell et al., 2016, Knickmeyer et al., 2010, McCormack et al., 2015; Mantini et al., 2011). The following Fig. 7, Fig. 8 demonstrate two examples of 21 consistent longitudinal ICNs identified via *LSDL*.

In addition, more details and slices of identified 21 consistent longitudinal ICNs across 4 macaques can be viewed by our website as follow:

1).http://hafni.cs.uga.edu/LSDL_Project/LSDL_Consistent/MonkeyBrain_Subject_1_LongitudinalVarianceComponentTemplatesMap_presentation.html

2).http://hafni.cs.uga.edu/LSDL_Project/LSDL_Consistent/MonkeyBrain_Subject_2_LongitudinalVarianceComponentTemplatesMap_presentation.html

3).http://hafni.cs.uga.edu/LSDL_Project/LSDL_Consistent/MonkeyBrain_Subject_3_LongitudinalVarianceComponentTemplatesMap_presentation.html

4).http://hafni.cs.uga.edu/LSDL_Project/LSDL_Consistent/MonkeyBrain_Subject_4_LongitudinalVarianceComponentTemplatesMap_presentation.html

Furthermore, for further quantitative validation of the 21 identified longitudinal consistent ICNs, we have conducted extensive quantitative analytics using voxel count on each ICN.

**Fig. 9:**
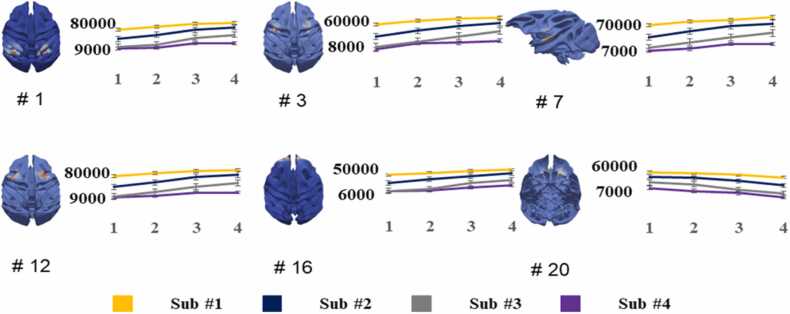
It illustrates a quantitative analysis of six representative ICNs, identified across four developmental stages: 3, 6, 12, and 18 months. These stages are denoted as Time Points 1, 2, 3, and 4, respectively. The analysis employs voxel count as a metric to measure the longitudinal variance within each ICN, offering insights into their developmental trajectories over time.

Overall, Fig. 9 illustrates a detailed quantitative analysis of six representative consistent longitudinal ICNs, highlighting changes in voxel counts from 3 to 18 months. The voxel count, a measure of activated voxels within each network, offers a precise indication of the changes in the area of each ICN over time. Specifically, the results reveal a consistent increase in the voxel count for ICNs #1, #3, #7, #12, and #16. This growth suggests a continuous expansion in the areas of the brain these ICNs cover, reflecting potentially significant developmental processes. The increase in voxel count indicates not just more extensive neural engagement within these networks but may also imply enhancements in functions associated with these areas, such as sensory integration, cognitive processing, and social interaction skills. In contrast, ICN #20 exhibits a gradual reduction in voxel count throughout the observed periods. This decline could indicate a process of synaptic pruning, where less efficient or less necessary neural connections are eliminated, potentially leading to a more efficient and specialized brain network. The consistent decrease in this network's area warrants further investigation to understand the developmental implications and underlying mechanisms. The contrasting trajectories observed among these ICNs underscore the dynamic and complex nature of brain development during early childhood. Notably, the reduction in ICN #20, amidst the growth observed in other networks, highlights an intriguing area for further research. Future studies should aim to include a larger cohort of macaques to validate these findings and explore the functional consequences of these developmental changes. Notably, ICNs, which represent the interaction between activated brain regions, typically reflect variations in deoxyhemoglobin concentration in rsfMRI data (Cui et al., 2022, Fiorenzato et al., 2019, Franzmeier et al., 2019). Consequently, the intensity for each individual reflects the specific activation levels within functional brain areas (Li et al., 2019, Peng et al., 2023, Yang et al., 2022). Given the uniqueness of each animal, the variation in intensity across individuals highlights these inherent differences.

Moreover, the detailed quantitative results and visualizations of this analysis can be accessed through our dedicated webpage:

http://hafni.cs.uga.edu/LSDL_Project/LSDL_Consistent/MonkeyHAFNILongitudinalDevelopmentTrajectory_presentation.html

On the dedicated webpage, we display representative slices of the corresponding ICN templates and the longitudinal voxel count data for the four macaque subjects involved in our experiments. Each subject is represented by a different color for clarity. Generally, the voxel counts of the longitudinal consistent ICNs show a continuous increase through all assessed time points, from 3 months to 18 months. This linear increase in voxel counts aligns with the developmental patterns reported in the research works by Machado, Malkova, and Knickmeyer (Howell et al., 2016, Knickmeyer et al., 2010, Machado and Bachevalier, 2003; Zhang et al., 2017; McCormack et al., 2015). The quantitative results effectively illustrate the linear variance, further validating the reliability and consistency of our findings in the developmental trajectory of these ICNs.

To summarize, these quantitative analyses not only provide valuable insights into the functional dynamics of early brain development but also set the stage for deeper explorations into how these changes impact overall brain function and development. Understanding these consistent and dynamic patterns is crucial for advancing our knowledge of neurological development and could have implications for early diagnosis and intervention in developmental disorders.

## 4 Discussion

In this work, we introduced an innovative *LSDL* method, specifically designed to unearth consistent longitudinal ICNs within macaque brains using functional MRI data collected over time. Through meticulous validation, *LSDL* has proven its superior efficacy by successfully identifying 21 consistent longitudinal ICNs, outperforming traditional methods such as FICA and ODL.

Overall, the effectiveness of *LSDL* was rigorously validated against several peer-reviewed methods, clearly demonstrating its superior ability to detect consistent longitudinal features—namely, ICNs. This not only underscores the robustness of *LSDL* but also showcases its potential to significantly advance research in critical areas such as adolescent neuroscience and developmental brain studies. The precision of *LSDL* paved the way for an in-depth investigation of functional developments in specific brain regions, thereby enhancing the accuracy and reliability of longitudinal studies.

In addition, our research extends beyond individual findings to encompass extensive comparative studies between adolescent primates and human infants, focusing on crucial areas like the visual cortex, auditory regions, and the Default Mode Network (DMN). These studies illuminate the similarities and differences in developmental patterns between species, enriched by the longitudinally consistent networks that *LSDL* identifies. Such insights are instrumental in understanding developmental trajectories and contribute profoundly to advanced comparative neuroscientific research.

Moreover, the consistent networks identified by *LSDL* can be integrated with a diverse array of biological data, including genetic, protein, connectivity, and anatomical information. This holistic approach is pivotal for refining the publicly available macaque longitudinal cortical parcellation atlas. It also pushes the boundaries of our understanding of brain development across different species, suggesting a comprehensive model of brain evolution that is deeply rooted in empirical evidence.

This study not only highlights the utility of the *LSDL* method in tracing and understanding longitudinal brain development but also sets a robust foundation for future explorations into the dynamic changes occurring in the brain from infancy through adulthood. By providing a sophisticated framework to reveal consistent longitudinal ICNs, *LSDL* significantly enhances our ability to decipher complex neurological trajectories and their implications for health and disease. Future research will leverage this advanced methodology to explore further the intricate processes underlying brain development and its disorders, opening new frontiers in neuroscience and clinical applications.

## Ethical approval

This study involved four subjects from a longitudinal cohort at the Yerkes National Primate Research Center (YNPRC) at Emory University, located in Lawrenceville, Georgia. Notably, the ethical considerations of this study were rigorously maintained, adhering to the Animal Welfare Act and the U.S. Department of Health and Human Services guidelines, with approval from the Emory University Institutional Animal Care and Use Committee (IACUC).

## Confidentiality

Participant confidentiality will be strictly maintained throughout the study. Data will be accessible only to the research team and will be used solely for the purposes of this study.

## Data protection

All data will be collected, stored, and processed in accordance with the relevant data protection laws and regulations, such as the General Data Protection Regulation (GDPR). Data will be securely stored in encrypted files and protected databases.

## Risk minimization

The study will be designed to minimize any potential risks to participants. Any foreseeable risks will be clearly communicated to the participants, and appropriate measures will be taken to mitigate these risks. In the event of any adverse effects, appropriate support and intervention will be provided.

## Conclusion

This statement reaffirms our commitment to conducting the proposed research with the highest ethical standards. We will ensure that all procedures are carried out with respect for the rights, dignity, and well-being of the participants.

## Conflict of interest and compliance with ethical standards

This Ethics Statement outlines the conflict of interest and ethical considerations and procedures that will be adhered to in the proposed research project. The study aims to 1). Propose and validate an effective Longitudinal Sparse Dictionary Leaning (LSDL) for fMRI cohorts’ analytics; 2). Present multiple consistent longitudinal intrinsic connectivity networks (ICNs) Ensuring the highest standards of ethical conduct is paramount throughout this research process.

## CRediT authorship contribution statement

**Wei Zhang:** Validation, Supervision, Resources, Methodology, Investigation, Funding acquisition, Data curation, Conceptualization. **Yu Bao:** Validation, Formal analysis. **Afrar Jahin:** Writing – review & editing, Writing – original draft, Conceptualization. **Arif Hassan Zidan:** Writing – review & editing, Writing – original draft.

## Conflict of Interest

All authors have no conflicts of interest to disclose.
